# Evaluation of Functional Selectivity of Haloperidol, Clozapine, and LASSBio-579, an Experimental Compound With Antipsychotic-Like Actions in Rodents, at G Protein and Arrestin Signaling Downstream of the Dopamine D_2_ Receptor

**DOI:** 10.3389/fphar.2019.00628

**Published:** 2019-06-04

**Authors:** Rafaela R. Silva, Lucas T. Parreiras-e-Silva, Thais E.T. Pompeu, Diego A. Duarte, Carlos A.M. Fraga, Eliezer J. Barreiro, Ricardo Menegatti, Claudio M. Costa-Neto, François Noël

**Affiliations:** ^1^Laboratory of Biochemical and Molecular Pharmacology, Institute of Biomedical Sciences, Federal University of Rio de Janeiro, Rio de Janeiro, Brazil; ^2^Laboratory of structure and function of 7 Transmembrane Receptors (7TMR), Department of Biochemistry and Immunology, Ribeirão Preto Medical School, University of São Paulo, Ribeirão Preto, Brazil; ^3^Laboratory of Evaluation and Synthesis of Bioactive Substances (LASSBio), Institute of Biomedical Sciences, Federal University of Rio de Janeiro, Rio de Janeiro, Brazil; ^4^Laboratório de Química Farmacêutica Medicinal, Pharmacy School, Federal University of Goiás, Goiânia, Brazil

**Keywords:** LASSBio-579, D2, schizophrenia, antipsychotics, β-arrestin, functional selectivity, biased agonism, clozapine

## Abstract

LASSBio-579, an *N*-phenylpiperazine antipsychotic lead compound, has been previously reported as a D_2_ receptor (D_2_R) ligand with antipsychotic-like activities in rodent models of schizophrenia. In order to better understand the molecular mechanism of action of LASSBio-579 and of its main metabolite, LQFM 037, we decided to address the hypothesis of functional selectivity at the D_2_R. HEK-293T cells transiently coexpressing the human long isoform of D_2_ receptor (D_2_LR) and bioluminescence resonance energy transfer (BRET)-based biosensors were used. The antagonist activity was evaluated using different concentrations of the compounds in the presence of a submaximal concentration of dopamine (DA), after 5 and 20 min. For both signaling pathways, haloperidol, clozapine, and our compounds act as DA antagonists in a concentration-dependent manner, with haloperidol being by far the most potent, consistent with its nanomolar D_2_R affinity measured in binding assays. In our experimental conditions, only haloperidol presented a robust functional selectivity, being four- to fivefold more efficient for inhibiting translocation of β-arrestin-2 (β-arr2) than for antagonizing Gi activation. Present data are the first report on the effects of LASSBio-579 and LQFM 037 on the β-arr2 signaling pathway and further illustrate that the functional activity could vary depending on the assay conditions and approaches used.

## Introduction

G protein-coupled receptors (GPCRs), also known as 7TM receptors (since they have seven hydrophobic transmembrane domains and do not always signal through G proteins), are the targets for the majority of drugs used therapeutically (Hauser et al., 2017), albeit a shift toward kinases and cytokines is occurring in drug discovery programs (Shih et al., 2018).

During the last 10 years, increasing evidences indicate that ligands for the 7TM receptors can be functionally selective, bearing differences in potency and/or intrinsic activity at different signaling pathways mediated by the same receptor (Urban et al., 2007). As ligands could be tailored for accentuating a beneficial cellular signal or reducing a debilitating signal, functional selectivity should be considered in drug discovery programs in an attempt to improve efficacy and/or reduce adverse effects (Kenakin, 2015). However, the capability to detect this property (also called biased agonism) could vary depending on the tools used, e.g., the subtype of receptor; the complement of intracellular signaling molecules such as G proteins, second messengers, β-arrestins, and G-protein-coupled receptor kinase (GRK); the approach for labeling the proteins; the assay conditions (concentration and nature of the agonist); the ligand-binding kinetics; and the kinetics intrinsic to different intracellular signaling events (Costa-Neto et al., 2016; Klein Herenbrink et al., 2016; Urs et al., 2017).

Such a 7TM receptor, the dopamine D_2_ receptor (D_2_R), is central for the treatment of schizophrenia since all antipsychotics in clinical use are able to block this receptor at therapeutical concentrations, albeit differences exist between first- and second-generation drugs. Indeed, whereas the first-generation (typical) antipsychotics are considered as relatively pure antagonists of D_2_R, the second-generation (atypical) antipsychotics are, usually, considered as multitarget drugs (Roth et al., 2004; Ginovart & Kapur, 2012). Classically, the D_2_Rs are coupled to G_i/o _proteins(canonical signaling pathway). However, Beaulieu et al. (2007) showed that when activated by dopamine, these receptors were able to regulate the Phosphatidylinositol 3-kinase/protein kinase–Glycogen synthase kinase-3 (Akt– GSK-3) pathway through a mechanism involving β-arrestin-2 (β-arr2) and independent of G protein (noncanonical signaling pathway). Furthermore, in addition to antagonizing the D_2_R-mediated G protein-dependent pathway, various clinically effective antipsychotics, either typical or atypical, have been reported as antagonists of the β-arr2 signaling pathway (Masri et al., 2008). On the other hand, aripiprazole has been reported to act as either a partial agonist or an antagonist of β-arr2 signaling, depending on the experimental conditions used (Allen et al., 2011; Koener et al., 2012). Recently, some *N*-phenylpiperazines have been reported as functionally selective for D_2_R-mediated pathways (Chen et al., 2016; Möller et al., 2017).

**Figure 1:**
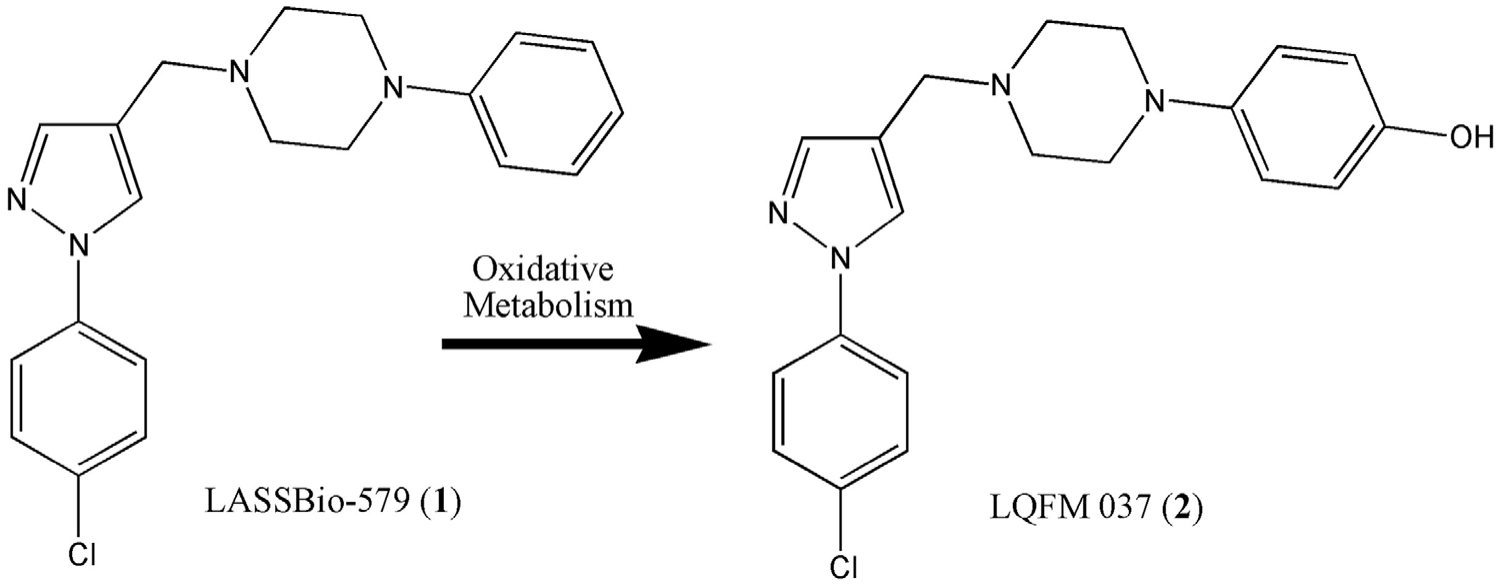
Structural pattern of multitarget heterocyclic *N*-phenylpiperazines (**1**) and (**2**).

We previously described the synthesis and pharmacological characterization of a series of heterocyclic *N*-phenylpiperazines resulting from hybridization and simplification of the lead compounds clozapine and L-741 (Menegatti et al., 2003; Neves et al., 2010). One of these compounds, LASSBio-579 [1-((1-(4-chlorophenyl)-1*H*-pyrazol-4-yl)methyl)-4-phenylpiperazine, **1**] (**Figure 1**), was orally active in different rodent models of positive and negative symptoms of schizophrenia (Neves et al., 2013) and had a moderate affinity for the D_2_, D_4_, and 5-hydroxytryptamin 1A (5-HT_1A_) receptors, with low affinity for the 5-hydroxytryptamin 2A (5-HT_2A_) receptor (Neves et al., 2010; Pompeu et al., 2013). The main phase I metabolite of LASSBio-579 in rats, identified as the *p*-hydroxylated derivative LQFM 037 (**2**) (**Figure 1**), was a ligand of the D_2_ and D_4_ receptors at submicromolar concentrations, indicating that it could participate in the *in vivo* effects of LASSBio-579 (Gomes et al., 2013). More recently, we showed that both LASSBio-579 and its metabolite were weak partial agonists of the D_2_R at the G-protein pathway and shared with clozapine the particularity to have rapid dissociation kinetics, contrary to haloperidol (Pompeu et al., 2015). We concluded that these two characteristics could explain, at least partially, the atypical-like profile previously reported when LASSBio-579 was administered to rodents. Here, we decided to address the hypothesis of functional selectivity at the D_2_R, using the BRET technique for measuring the effect of both LASSBio-579 and LQFM-037 on the β-arr2 and G_i_-dependent signaling pathways.

## Materials and Methods

Haloperidol, dopamine hydrochloride, and (−)-quinpirole hydrochloride were purchased from Sigma-Aldrich (São Paulo, Brazil). Clozapine was purchased from Tocris Bioscience. LASSBio-579 and LQFM 037 were synthesized and characterized as previously described (Neves et al., 2010; Gomes et al., 2013). The test substances and (−)-sulpiride were dissolved in dimethyl sulfoxide (DMSO) to obtain stock solutions. Dopamine and quinpirole were dissolved in ultrapurified water. All subsequent dilutions were prepared in water. At the final concentration used (below 0.5%), DMSO had no effect in our assays.

### Binding Experiments

We performed receptor-binding assays in a rat (total) striatum preparation to determine the affinity of haloperidol for D_2_R, as previously described for the other compounds used here (Gomes et al., 2013). Adult male Wistar rats (200–300 g) were killed by decapitation, their brains were immediately removed on ice, and the striatum was dissected and stored in liquid nitrogen until use. This procedure was approved by the Institutional Ethical Committee for Animal Care from the Federal University of Rio de Janeiro (no. 01200.001568/2013-87).

Striatum was homogenized in a Potter apparatus with a motor-driven Teflon pestle at 4°C in 20 volumes per gram of tissue of ice-cold Tris-HCl 50 mM buffer (pH 7.4) containing MgCl_2_ 8 mM and Ethylenediaminetetraacetic acid (EDTA) 5 mM. The resulting suspension was ultracentrifuged (48,000 g_av_ at 4°C) for 20 min. The pellet was resuspended in 20 volumes of buffer and incubated at 37°C for 10 min for removal of endogenous neurotransmitters. This suspension was cooled on ice and ultracentrifuged twice (48,000 g_av_ for 20 min at 4°C). The final pellet was resuspended in buffer, yielding a proportion of 1.5 mL/g tissue and stored in liquid nitrogen until use. The protein concentration was determined by the method of Lowry et al. (1951) using bovine serum albumin as standard.

Striatal membranes from rat brain (50 µg protein) and 0.1 nM [^3^H]-YM-09151-2 (82.5 Ci/mmol, New England Nuclear) were incubated at 37°C for 60 min under yellow light in a buffer solution of Tris–HCl 50 mM (pH 7.4) containing 120 mM NaCl, 5 mM KCl, 5 mM MgCl_2_, 1.5 mM CaCl_2_, and 1 mM EDTA, in a final volume of 500 µL, in the absence or presence of different concentrations of haloperidol. The nonspecific binding was estimated using 30 μM (−) sulpiride (Tocris Bioscience). After incubation, samples were rapidly diluted with 3 × 4 mL of cold buffer Tris–HCl 5 mM (pH 7.4) and immediately filtered under vacuum on glass fiber filters (GMF 3, Filtrak, Germany) previously soaked in the same buffer. Filters were then dried and immersed in a scintillation mixture, and the radioactivity retained in the filters was counted with a Packard Tri-Carb 1600 TR liquid scintillation analyzer (PerkinElmer).

Competition curves with haloperidol were performed, and mean inhibitory concentrations (IC_50_) were estimated from the competition binding curves by nonlinear regression using the classical equation for simple bimolecular reaction (one site competition, GraphPad Prism version 6.0), assuming a single population of binding sites. The *K*_i_ value was calculated from the Cheng–Prusoff equation: *K*_i_ = IC_50_/[1 + (radioligand)/*K*_d_]. The *K*_d_ value used for [^3^H]-YM-09151-2 (0.42 nM) was obtained from saturation experiments conducted in our preparation.

### Cell Culture and Transfection

HEK293T cells were cultured in Dulbecco’s modified eagle medium (DMEM) supplemented with 10% fetal bovine serum and 100 U/mL penicillin/streptomycin at 37°C in 5% CO_2_. Forty-eight hours before the experiments, cells were transfected in suspension, using polyethylenimine (PEI; 25 kDa linear; Polysciences, Warrington, PA, USA) at a ratio of 3:1 PEI/DNA, and directly seeded in 96-well white plates (OptiPlate; PerkinElmer) at a density of 4 × 10^4^ cells/well and at 37°C in 5% CO_2_. When needed, total DNA amount was adjusted with salmon sperm DNA (Invitrogen, Carlsbad, CA, USA).

### Analysis of G Protein Activation and β-Arrestin Translocation by Bioluminescence Resonance Energy Transfer Assays

For assessment of G_i_ activation by D_2_R using BRET, HEK293T cells were transiently transfected with plasmids encoding D_2L_R, G_iα3_-RLucII, untagged Gβ1, and GFP10-Gγ1 and stimulated with dopamine for 5 or 20 min as described before (Corrêa et al., 2017). For measurement of β-arrestin translocation to plasma membrane by enhanced bystander BRET (ebBRET) (Namkung et al., 2016), cells were transfected with plasmids encoding D_2L_R, RLucII-β-arrestin2, and rGFP-CaaX, and stimulated with dopamine for 5 or 20 min. BRET values were monitored using Victor™ X Light Luminescence microplate reader (Perkin Elmer) equipped with BRET400-GFP2/10 filter set (acceptor, 515 ± 20 nm; and donor, 400 ± 70 nm filters), 5 min after the addition of 5 μM of coelenterazine 400-a (Biotium, Hayward, CA, USA). In the antagonism assays, the different ligands were simultaneously incubated with dopamine, using a submaximal concentration of dopamine and increasing concentrations of the evaluated ligands.

### Evaluation of Functional Selectivity at the Dopamine D_2_ Receptor

To assess the functional selectivity of the drugs, we first had to calculate pseudo-*K*_i_ values (usually referred as *K*_B_ in functional studies) in order to compare potencies measured in the two assays since they were performed with different concentrations of the agonist dopamine (exhibiting different potencies in the two assays). Accordingly, we corrected the IC_50_ values obtained in the nonsteady-state conditions, using the Cheng–Prusoff equation, classically used for equilibrium competition binding assays and mathematically equal to the one used by Masri et al. (2008) in similar conditions. Then, for every compound, we calculate the ratios of their *K*_i_ and *I*_max_ values for the two pathways.

Alternatively (supplementary data), as the *E*_max_ values were different for some drugs, we also used the hybrid parameter *I*_max_/*K*_i_ for assessing the “efficiency” of the drug to block each pathway activated by dopamine. The *I*_max_ values were expressed as percentage of the dopamine effect that was blocked in the experimental condition used in each of the two BRET assays.

## Results

### *In Vitro* Binding Assays

**Figure 2:**
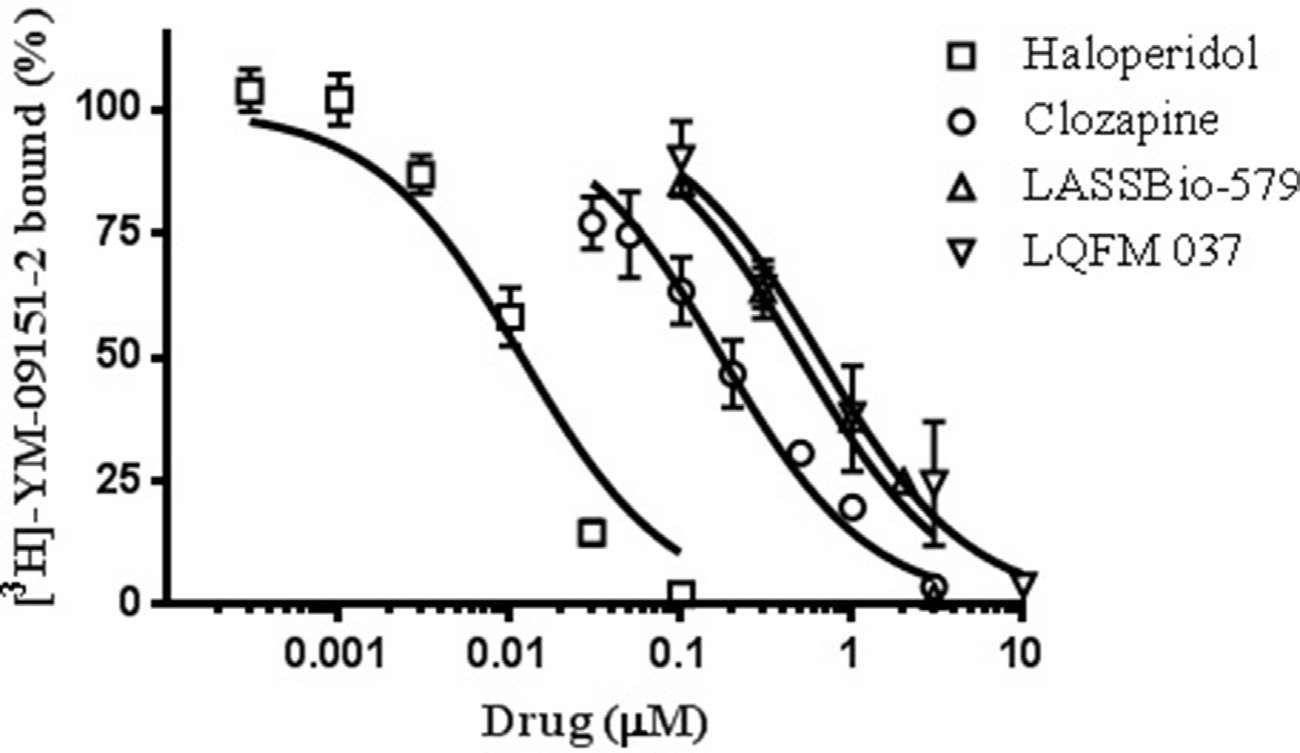
Effect of compounds on [^3^H]-YM-09151-2 binding to D_2_ receptor (D_2_R) present in rat striatum membrane preparation. Data are means (±S.E.) from two to four independent experiments, each performed in triplicate. The data were fitted assuming a single population of binding sites, and curves were drawn using the parameters fitted by nonlinear regression (“one site - Fit logIC50,” GraphPad Prism version 6.0®).

Affinity of haloperidol for D_2_-like receptors was determined *via* standard competition assays using rat striatum homogenate with [^3^H]-YM-9151-2 as the radioactive ligand (**Figure 2**). The affinities of the other compounds were previously determined in the same experimental conditions (Gomes et al., 2013). The results of these assays are shown in **Figure 2** and **Table 1**.

**Table 1:** Apparent affinities (*K*_i_) and maximal effect (*I*_max_) of the ligands for inhibition of dopamine-induced G_i _activation, β-arrestin-2 (β-arr2) translocation, and binding to D_2_ receptor (D_2_R).

Signaling pathways	*K_i_* (nM) (95% CI)			
	Haloperidol	Clozapine	LASSBio-579	LQFM 037
5 min G_i_	6.36 (2.50–16.2)	393 (86–1791)	118 (16–864)	263 (47–1479)
5 min β-arr	1.57 (0.83–2.97)	329 (146–741)	109 (41–291)	353 (86–1441)
20 min G_i_	4.65 (1.67–13.0)	303 (74–1228)	66.7 (17.3–257)	190 (44–811)
20 min β-arr	0.89 (0.48–1.65)	261 (140–486)	41.6 (18.1–95.5)	209 (102–429)
Binding D_2_R (rat)	10	130	380	530
	*I*_max_ (% dopamine effect) ± S.E.M.			
5 min G_i_	104 ± 8	108 ± 24	75.1 ± 16.3	78.9 ± 19.2
5 min β-arr	104 ± 6	128 ± 13	148 ± 20	71.3 ± 12.3
20 min G_i_	111 ± 10	108 ± 20	72.4 ± 9.8	71.6 ± 13.7
20 min β-arr	104 ± 6	138 ± 10	129 ± 12	94.2 ± 7.4

The IC_50_ values obtained in the nonsteady-state conditions were corrected using the Cheng–Prusoff equation in order to calculate the apparent affinities (K_i_). For the competition binding assay performed at equilibrium, the IC_50_ values were corrected using the Cheng–Prusoff equation. The value for haloperidol was obtained in the same experimental conditions as described previously for clozapine, LASSBio-579, and LQFM 037 (Gomes et al., 2013 and Materials and Methods).

### Intrinsic Activity for D_2 _Receptor-Mediated G Protein Signaling

**Figure 3:**
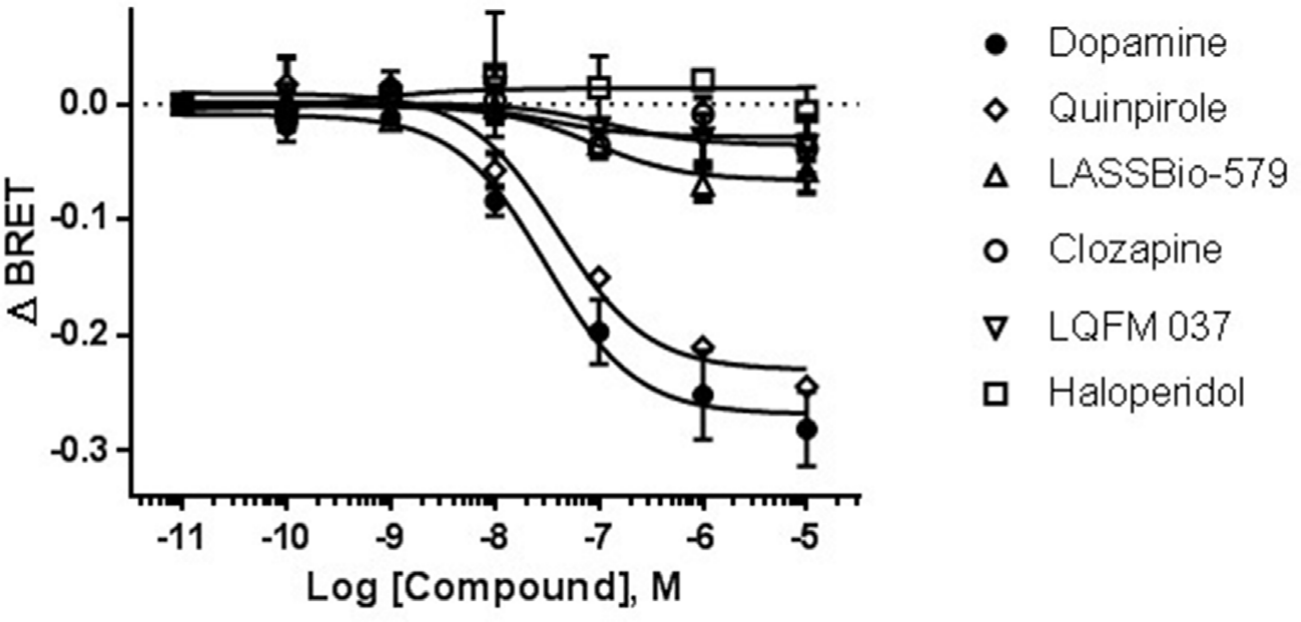
Concentration–effect curves for the G_i_ activation (agonistic effect at the D_2_R canonical pathway). Bioluminescence resonance energy transfer (BRET) signal measured after 5 min of stimulation of HEK-293T cells as described in Materials and Methods. Data are means (±S.E.) of three independent experiments, each performed in triplicate.

According to the canonical signaling pathway, activation of D_2_R leads to G_i_ protein activation and separation of the G_α_ subunit from the G_βγ_ subunits. This event was detected here as a decrease in the BRET signal. As shown in **Figure 3**, dopamine induced a concentration-dependent G_i_ activation [EC_50_ = 31 nM; *E*_max_ = −0.27, 95% CI: (−0.30; −0.24)]. Using the same system, quinpirole was also a potent full agonist [EC_50_ = 40 nM; *E*_max_ = −0.23, 95% CI: (−0.25; −0.21)]. On the other hand, the typical antipsychotic haloperidol had no effect [*E*_max_ = 0.01, 95% CI: (−0.014; 0.040)], whereas clozapine (used as reference for the atypical antipsychotics) and our compounds presented a small, but statistically significant, effect with *E*_max_ values (95% CI): −0.03 (−0.045; −0.012), −0.07 (−0.085; −0.047), and −0.04 (−0.062; −0.009) for clozapine, LASSBio-579, and LQFM 037, respectively.

### Antagonist Activity for D_2_ Receptor-Mediated G Protein Signaling

**Figure 4:**
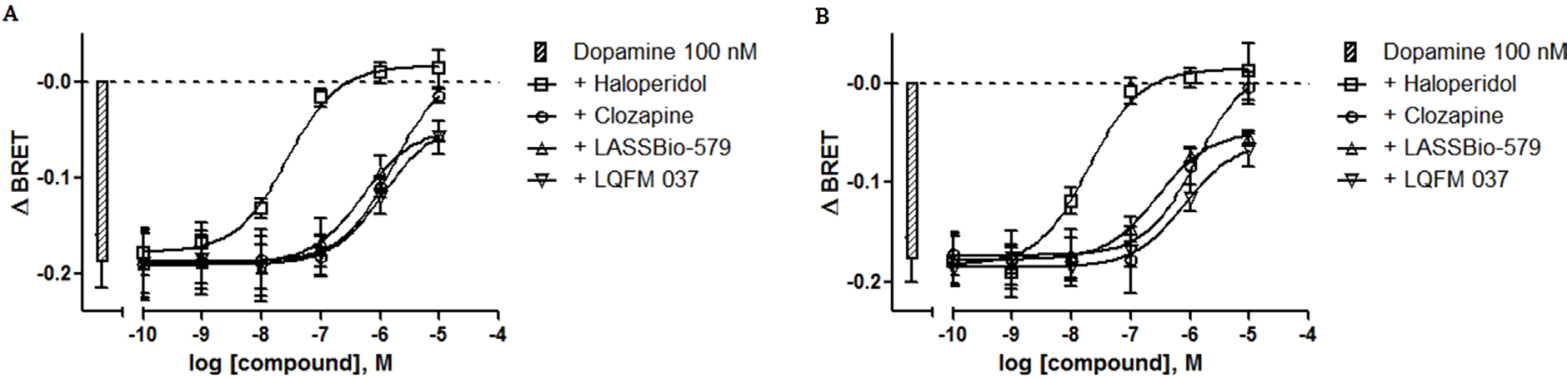
Concentration–effect curves for the inhibition of dopamine-induced G_i_ activation. BRET assay was performed after 5-min **(A)** or 20-min **(B)** stimulation of HEK-293T cells with compounds in the presence of 100 nM dopamine, as described in Materials and Methods. Data are means (±S.E.) of four independent experiments, each performed in triplicate.

In order to evaluate the antagonist activity with respect to the dopamine effect on G_i_, we used the same protocol but incubating different concentrations of the compounds in the presence of 100 nM dopamine, a sub-maximal concentration of this full agonist (see **Figure 3** and control in **Figure 4**), for 5 and 20 min. The results (**Figure 4A and B**) showed that all four compounds act as antagonists in a concentration-dependent manner after 5- and 20-min incubation, with haloperidol being by far the most potent. The values of IC_50 _obtained by nonlinear regression analysis of these data were used to calculate the apparent affinities reported in **Table 1**. Note that LASSBio-579 and LQFM 037 did not fully inhibit the effect of dopamine, with *I*_max_ values around 70–80% (**Table 1**) as expected for (weak) partial agonists.

### Intrinsic Activity for D_2 _Receptor-Mediated β-Arrestin-2 Translocation

**Figure 5:**
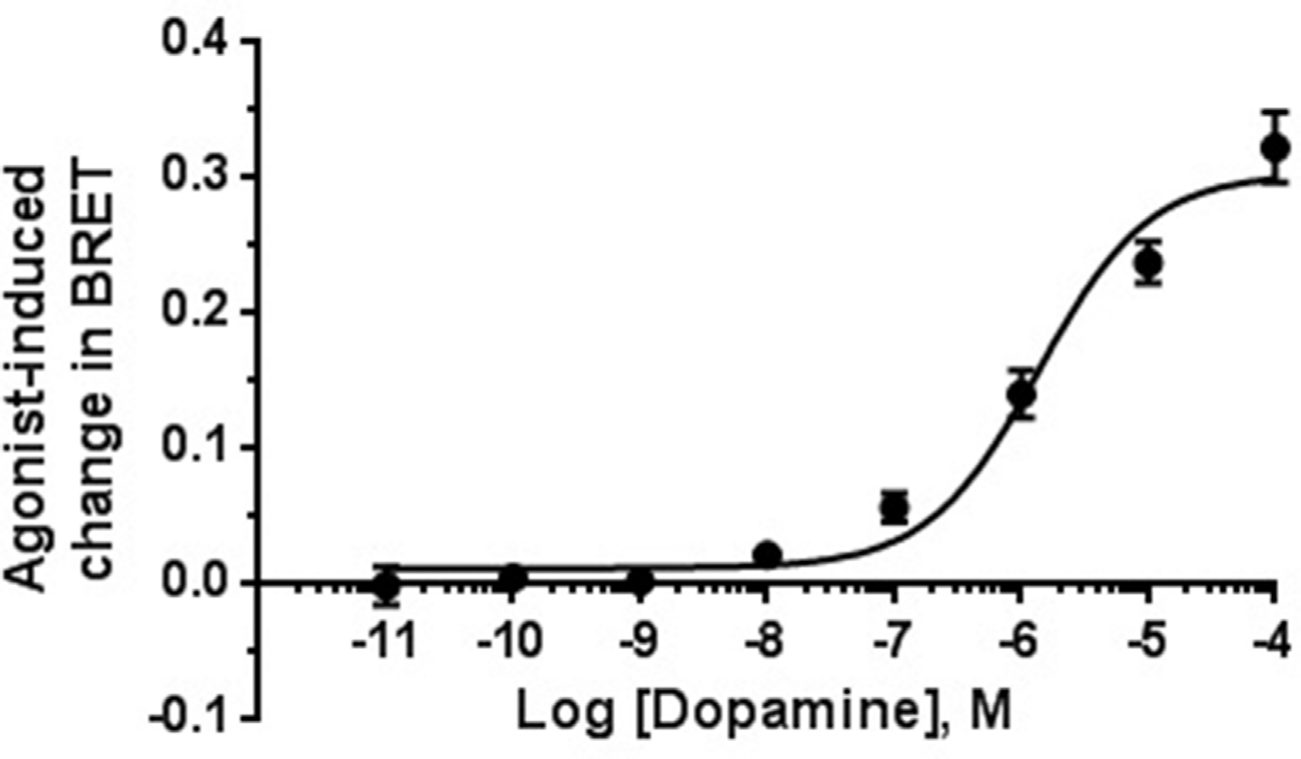
Concentration–effect curve for dopamine-induced translocation of β-arrestin-2. BRET signal measured 20 min after stimulation of HEK-293T cells as described in Materials and Methods. Data are means (±S.E.) of two independent experiments, each performed in triplicate.

**Figure 6:**
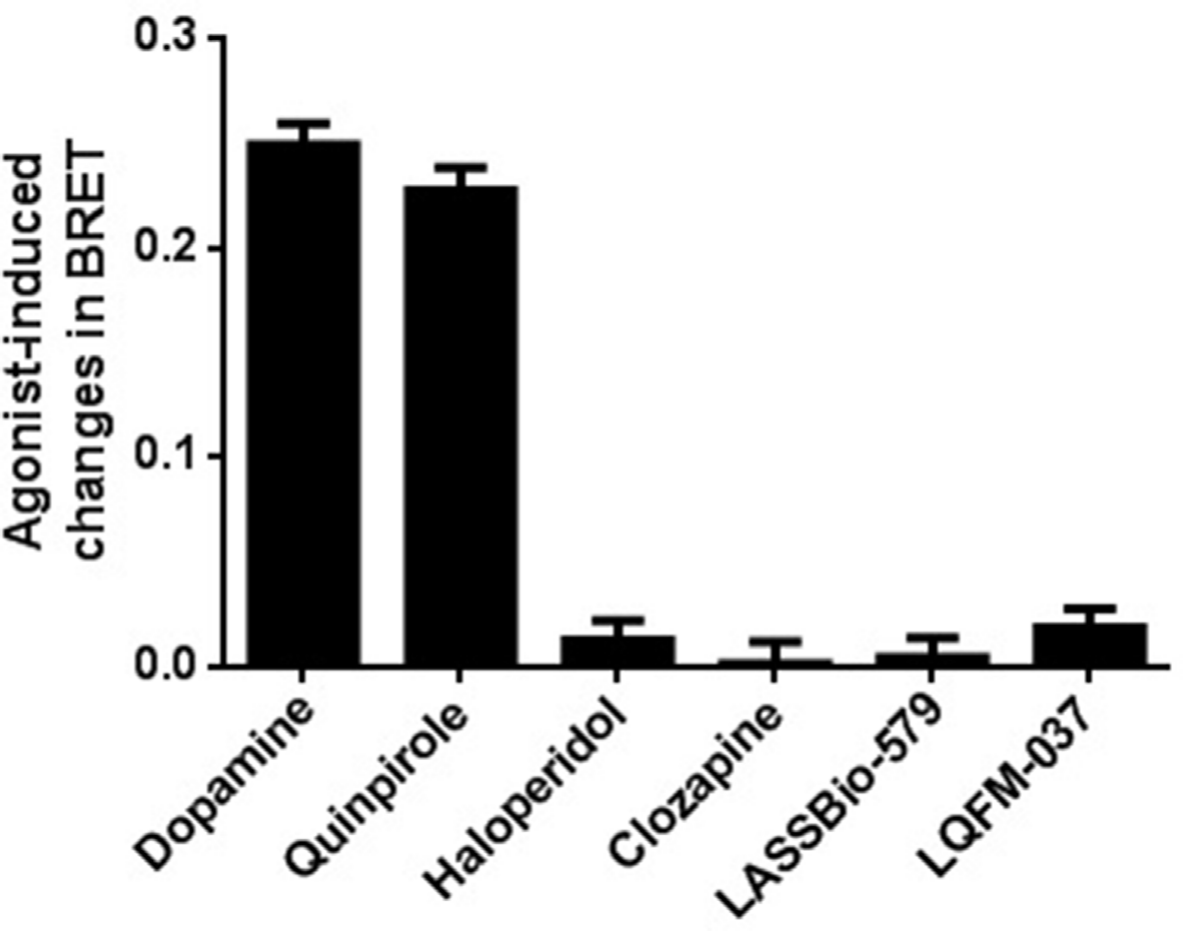
Drug-induced change in BRET for translocation of β-arrestin-2. A relatively high concentration of each drug (10 µM) was used for comparing their agonistic effect. BRET signal was measured 20 min after stimulation of HEK-293T cells as described in Materials and Methods. Data are means (±S.E.) of two independent experiments, each performed in triplicate.

As shown in **Figure 5**, stimulation with the endogenous agonist dopamine induced a concentration-dependent translocation of β-arr2 [EC_50_ = 1.4 µM; 95% CI: (0.54; 3.4 µM)]. Contrary to the classical D_2_R agonists (dopamine and quinpirole), none of the compounds was able to induce β-arr2 translocation even at the high concentration tested (10 µM) (**Figure 6**).

### Antagonist Activity for D_2 _Receptor-Mediated β-Arrestin-2 Translocation

**Figure 7:**
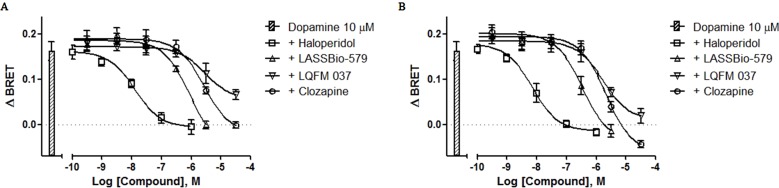
Concentration–effect curves for the inhibition of dopamine-induced translocation of β-arrestin-2. BRET assay was performed after 5-min **(A)** or 20-min **(B)** stimulation of HEK-293T cells with compounds and 10 μM dopamine, as described in Materials and Methods. Data are representative (means ± S.E.) of four independent experiments, each performed in triplicate.

We also determined the ability of each compound to antagonize the β-arr2 translocation induced by dopamine (10 µM) after 5- and 20-min incubation. All four compounds block the effect of dopamine in a concentration-dependent manner, although great differences exist with respect to their potencies with haloperidol being by far the most potent (**Figure 7A and B**). The values of IC_50 _obtained by nonlinear regression analysis of these data were used to calculate the apparent affinities reported in **Table 1**.

### Functional Selectivity of Compounds for D_2L_R Canonical and Noncanonical Signaling Pathways

To assess the functional selectivity of the drugs, we had to calculate pseudo-*K*_i_ values (usually referred as *K*_B_ in functional studies) in order to compare efficiencies measured in the two assays, since they were performed with different concentrations of the agonist dopamine (exhibiting different potencies). Accordingly, we corrected the IC_50_ values obtained in the nonsteady-state conditions (see Materials and Methods) using the Cheng–Prusoff equation. The IC_50_ values were divided by 4.2 and 8.1 for G_i_ and β-arr2, respectively. Based on the ratios of *K*_i_ values for the two pathways (**Table 2**), only haloperidol presents some functional selectivity, with a *K*_i_ (Gi/β-arr) ratio around 4–5, being more potent for antagonizing the β-arrestin pathway. Note that the data were very similar when measured after 5 or 20 min.

**Table 2:** Estimation of functional selectivity. The ratio of potency (*K*_i_) and efficacy (*I*_max_) for each pathway was used for estimating the presence of functional selectivity.

Time	Haloperidol	Clozapine	LASSBio-579	LQFM 037
***K*_i (Gi)_/*K*_i (β-arr)_**				
5 min	4.05	1.19	1.08	0.74
20 min	5.22	1.16	1.60	0.91
***I*_max (Gi)_/*I*_max (β-arr)_**				
5 min	1.00	0.84	0.51	1.11
20 min	1.07	0.78	0.56	0.76

**Table 2** also presents separately for every compound the ratios of their *I*_max_ values for the two pathways.

## Discussion

The present study aimed to better understand the molecular mechanism of action of LASSBio-579, our lead compound rationally designed through hybridization of clozapine and L-741 (Menegatti et al., 2003), that was previously reported as a new atypical antipsychotic candidate. We addressed the hypothesis of functional selectivity at the dopamine D_2_R by comparing the effect of LASSBio-579 and its main phase I metabolite (LQFM 037) on the β-arrestin-2 (β-arr2) and G_i_ signaling pathways. For this purpose, we developed two different BRET assays, based on HEK293T cells expressing the human D_2_R (variant 1). In addition, we performed assays with haloperidol (used as reference for the typical antipsychotics) and clozapine (used as reference for the atypical antipsychotics), as already performed by others (Masri et al., 2008; Möller et al., 2017).

In order to compare the present data of haloperidol binding with previous data of the other compounds, we had to use exactly the same experimental conditions (Gomes et al., 2013). Nevertheless, it is important to highlight that we performed receptor-binding experiments in the total striatum, a structure that contains both D_2_R and D_3_R. However, the density of our target D_2_R was estimated to be higher (twofold) compared to D_3_R (Booze and Wallace, 1995). Furthermore, since the competition curves were well fitted by the model considering a single population of receptors (Hill coefficient = 1), either the contribution of the D_3_R was too small or the drugs have similar affinities for both subtypes of receptors.

Regarding the ligand-dependent coupling of G_i_ protein, our BRET assay showed that LASSBio-579 and LQFM 037, in a smaller extent, exhibited a (very) weak intrinsic activity. This finding is in agreement with our previous data using a [^35^S]-GTPγS binding assay and a membrane preparation of Hela Tet-On cells overexpressing the mouse D_2L_R. Indeed, we reported that LASSBio-579 and LQFM 037, like aripiprazole but not clozapine, were weak partial agonists of the D_2_R when using such assay, especially in the absence of sodium ions in the incubation medium (Pompeu et al., 2015). These experimental conditions were previously shown to increase the sensitivity to detect weak partial agonists such as aripiprazole, since sodium ions disfavor the formation of the ternary complex of GPCRs (Lin et al., 2006). On the contrary, haloperidol, the reference typical antipsychotic, had no effect on G_i_ coupling. Our results are compatible with a previous report that clozapine and haloperidol failed to produce any detectable changes in [^35^S]-GTPγS binding and forskolin-stimulated cyclic adenosine monophosphate (cAMP) accumulation in Chinese hamster ovary (CHO) cells expressing the human D_2L_R (Jordan et al., 2007). On the other hand, Masri et al. (2008) showed that clozapine and haloperidol acted as inverse agonists of the G_i_ pathway using HEK293 cells stably expressing D_2L_R and exchange protein activated by cAMP (EPAC) biosensor, since they increased the forskolin-stimulated cAMP accumulation. Such results were also reported by others for clozapine (Akam & Strange, 2004) and haloperidol (Nilsson & Eriksson, 1993), but only in certain specific conditions (using T343R mutant D_2_R that is prone to adopt an active conformation in the absence of the agonist, being more sensitive to the effects of inverse agonists, or with very high haloperidol concentrations, allowing a nonspecific effect, unrelated to D_2_R). As a result, we conclude that the discrepancies across the studies are due to the different experimental conditions used.

We next addressed the capacity of the compounds to antagonize the dopamine-induced G_i_ activation after 5- and 20-min incubation. Independently of the incubation time, we observed that all compounds act functionally as antagonists, in a concentration-dependent manner. However, haloperidol displayed a markedly higher potency, consistent with its nanomolar D_2_R binding affinity. At the highest concentrations, haloperidol completely blocked the effect of dopamine, in accordance with its absence of intrinsic activity. Under the same conditions, clozapine also acted as a functional antagonist of dopamine at the canonical signaling pathway, indicating that the very weak intrinsic activity detected when it was used alone is functionally irrelevant. LASSBio-579 and LQFM 037 also antagonized the effect of dopamine in a concentration-dependent manner, but we were not able to obtain complete inhibition curves due to their lack of solubility at higher concentrations. However, the estimated *I*_max _are not maximal, as expected for weak partial agonists, as we observed for blocking of [^35^S]-GTPγS binding stimulation by dopamine (Pompeu et al., 2015).

As clinically effective antipsychotics have been shown to prevent dopamine-induced recruitment of β-arr2 (Masri et al., 2008), we designed a BRET assay with enough sensibility to quantitatively measure the effect of compounds at this noncanonical D_2_R pathway. In this recently developed BRET assay system, β-arr2 translocation to the membrane increases the BRET signal. In our conditions, none of the compounds exhibited intrinsic activity for this endpoint. These results are in accordance with previous data for clozapine and haloperidol (Klewe et al., 2008; Masri et al., 2008), whereas the data of our compounds LASSBio-579 and LQFM 037 are original. Using the same BRET-based assay, we showed that all four compounds block the effect of dopamine (10 µM) on β-arr2 translocation in a concentration-dependent manner, with haloperidol being the most potent. Note that the *I*_max_ values for clozapine and LASSBio-579 (above 100%) are probably not very robust since these compounds did not present inverse agonism when used alone (**Figure 6**).

Among the antipsychotics in clinical use tested for recruitment of β-arr2, only aripiprazole appeared as a partial agonist, as reported by Klewe et al. (2008). This activity was also reported by Allen et al. (2011) in special systems with CHO cells (using the DiscoveRx approach for measurement) or HTLA cells, a HEK293-derived cell line stably expressing β-arrestin and that is transfected with a D_2_R construct containing V2 vasopressin receptor tail, described as “Tango”-type assay. In the same study, Allen et al. (2011) showed that aripiprazole was also a partial agonist for β-arr2 signaling pathway in HEK293T cells coexpressing D_2_R and β-arr2, but only if G-protein-coupled receptor kinase 2 (GRK2) was also coexpressed. As GRK promotes the phosphorylation of GPCR that mediates the recruitment of the scaffolding protein β-arrestin, the level of GRK2 expression influences the drug capability to exhibit some intrinsic activity. As for the G_i_ activation assay, additional variables can influence the results such as sensibility of the assay, type of cells, and experimental conditions, e.g., time course.

Finally, we addressed the hypothesis of functional selectivity at the D_2_R, by comparing the potencies of compounds for blocking G_i_ activation induced by dopamine and dopamine-induced translocation of β-arr2. Since both assays were performed with different concentrations of the agonist dopamine (since it exhibited different potencies for these effects), it was necessary to correct the IC_50_ for calculating *K*_i _values. Although the Cheng–Prusoff equation is classically applied for equilibrium competition binding assays, we used it here for our nonsteady-state conditions, as already performed by others (Masri et al., 2008). Note that the compounds were added with the agonist concomitantly, as also performed by Masri et al. (2008) and Möller et al. (2017).

The Cheng–Prusoff equation could be applied to previous data reported by Möller et al. (2017) in order to obtain a value of *K*_B_ (we prefer the *K*_i_ terminology for antagonists) for haloperidol (≈0.6 nM) at the β-arr2 pathway very similar to ours, with the same type of cell but different agonists and approaches. Applying an equation mathematically identical to Cheng–Prusoff when Hill coefficient is equal to 1, Masri et al. (2008) obtained a *K*_B_ value for clozapine (0.07 μM) similar to ours for the β-arr2 recruitment after 20-min incubation. They used the same type of cell and receptor, but another agonist and different approaches for labeling the proteins and to detect their interactions.

In our experimental conditions, clozapine had no functional selectivity whereas haloperidol was four- to fivefold more potent for inhibiting the noncanonical pathway than for antagonizing G_i_ activation. Masri et al. (2008) reported that haloperidol was threefold more potent to block β-arr2 recruitment than to block adenylyl cyclase inhibition induced by a D_2_R agonist, in agreement with our data. On the other hand, these authors showed that clozapine was highly functionally selective, since it was 100-fold more potent to inhibit the noncanonical pathway than to antagonize the canonical pathway. The discrepancy between this study and our data could be due to the different intracellular signaling molecules analyzed (G proteins or second messengers) and the tools used, indicating that we have to be cautious in order to not generalize conclusions from a particular study, since numerous experimental conditions can influence the results. Note that kinetics aspects (Costa-Neto et al., 2016) do not seem to be an explanation here, since we obtained similar results after 5- or 20-min incubation (Costa-Neto et al., 2016).

Based solely on the *K*_i_ values for the two pathways, our compounds did not display any functional selectivity. As their *I*_max_ values were apparently lower for the G_i_ pathway than for the β-arrestin pathway, we felt interesting to also propose an alternative way for estimating functional selectivity in such a case, based on the hybrid *I*_max_/*K*_i_ for assessing the “efficiency” of the drug to block each pathway activated by dopamine. As shown in the supplementary material (**Table S1**), albeit such analysis could indicate a small tendency of functional selectivity for LASSBio-579, such property was only confirmed for haloperidol, with ratios around 4–5, indicating a higher capacity to antagonize the DA effect through the β-arrestin pathway than the G_i_ pathway.

As LASSBio-579 and LQFM 037 are *N*-phenylpiperazines, it is relevant to comment on a series of such compounds obtained by modification of the aripiprazole scaffold (Allen et al., 2011). One of these, UNC9994, presented an impressive functional selectivity for β-arr2 recruitment when compared to the G_i_ signaling pathway. Indeed, this compound acted as a partial agonist for β-arr2 recruitment, in special systems (with GRK2 coexpression or DiscoveRx and “Tango”-type assays), whereas it acted as an antagonist at the G_i_ signaling pathway. However, this result could not be replicated by a recent paper from an independent laboratory (Ågren et al., 2018). Another study reported great differences of functional selectivity at D_2_R among *N*-phenylpiperazines of similar chemical structures, with some favoring activation of G proteins (preferably G_o_) over β-arrestin recruitment, indicating that understanding such structure–activity relationship could open the way for designing drugs with the desired selectivity (Möller et al., 2017).

In summary, our results reveal a small degree of functional selectivity for haloperidol in D_2_R, but not for clozapine and our test compounds. The inverse agonism activity of clozapine and haloperidol in the G_i_ pathway and the pronounced functional selectivity of clozapine reported by others were not observed in our work, reinforcing the idea that functional activity can also be affected by assay conditions and used approaches (for a review, see Pupo et al., 2016).

## Ethics Statement

This study was carried out in accordance with the “Diretriz Brasileira para o Cuidado e a Utiliza de Animais para fins Cientificos e Didaticos—DBCA” from “Conselho Nacional de Controle de Experimenta Animal—CONCEA.” The protocol was approved by Institutional Ethical Committee for Animal Care from the Federal University of Rio de Janeiro (no. 01200.001568/2013-87).

## Author Contributions

RS: Conducted experiments; performed data analysis; wrote the manuscript. LP: Participated in research design; produced experimental tools; conducted experiments; performed data analysis; contributed to the manuscript. TP: Conducted experiments; performed data analysis. DD: Conducted experiments; performed data analysis. CF: Designed and contributed to the synthesis of LASSBio-579. EB: Contributed to the molecular design of LASSBio-579. RM: Participated in the design and synthesis of LQFM 037. CC-N: Participated in research design; contributed to the manuscript. FN: Participated in research design; contributed to the manuscript.

## Funding

This study was supported by Instituto Nacional de Ciência e Tecnologia de Fármacos e Medicamentos (INCT-INOFAR), by Fundação de Amparo à Pesquisa do Estado do Rio de Janeiro (FAPERJ) Grants Nos. E-26200.861/2018 and E-26/202.993/2016, by Conselho Nacional de Desenvolvimento Científico e Tecnológico (CNPq) Grants Nos. 465.249/2014-0 and 301650/2013-6, and by Fundação de Pesquisa do Estado de São Paulo (FAPESP) Grant 2012/20148-0. RS thanks FAPERJ for the scholarships. DD and LP are recipients of FAPESP scholarships. EB is supported by CNPq 304.187/2016-0.

## Conflict of Interest Statement

The authors declare that the research was conducted in the absence of any commercial or financial relationships that could be construed as a potential conflict of interest.
