# Supplementary material for: Evaluation of Functional Selectivity of Haloperidol, Clozapine, and LASSBio-579, an Experimental Compound With Antipsychotic-Like Actions in Rodents, at G Protein and Arrestin Signaling Downstream of the Dopamine D2 Receptor
Source: Front Pharmacol. 2019 Jun 4;10:628. doi: 10.3389/fphar.2019.00628 (PMC6558205; doi:10.3389/fphar.2019.00628)
Supplement: Supplementary file 1 [file Table_1.doc]

Supplementary material

Table 1. Estimation of functional selectivity. The drug efficiency for blocking the dopamine effect on each pathway was estimated by the hybrid parameter (I_max_ / *K*_i_) considering both the efficacy (I_max_) and the potency (*K*_i_). The ratio of these hybrid parameters for each pathway was as a tool for estimating the presence of functional selectivity.

|  | **(I_max_ / *K*_i_)** _β-arr_ **/ (I_max_ / *K*_i_)** _Gi_ | | | |
| --- | --- | --- | --- | --- |
| **signaling** **pathways** | **Haloperidol** | **Clozapine** | **LASSBio-579** | **LQFM 037** |
| 5 min β-arr / 5 min G_i_ | 4.04 | 1.45 | 2.11 | 0.67 |
| 20 min β-arr / 20 min G_i_ | 4.91 | 1.48 | 2.79 | 1.19 |
